# Detection of Autoantibodies in Saliva as New Avenue for the Diagnosis and Management of Autoimmune Patients

**DOI:** 10.3390/diagnostics12082026

**Published:** 2022-08-22

**Authors:** Savino Sciascia, Chelsea Bentow, Massimo Radin, Alice Barinotti, Irene Cecchi, Silvia Foddai, Dario Roccatello, Michael Mahler

**Affiliations:** 1 University Center of Excellence on Nephrologic, Rheumatologic and Rare Diseases (ERK-Net, ERN-Reconnect and RITA-ERN Member) with Nephrology and Dialysis Unit and Center of Immuno-Rheumatology and Rare Diseases (CMID), Coordinating Center of the Interregional Network for Rare Diseases of Piedmont and Aosta Valley, San Giovanni Bosco Hub Hospital, 10154 Turin, Italy; 2 Werfen Autoimmunity, San Diego, CA 92131, USA

**Keywords:** autoantibodies, antinuclear antibodies, autoimmune disease, saliva

## Abstract

(1) Background: Autoimmune diseases are characterized by autoantibodies directed to a large number of antigenic targets and are measured using serum as sample matrix. Although serum is a very common specimen type, it comes with certain drawbacks. Most importantly, it depends on venous puncture and requires medical personnel for sampling. This is of particular importance in light of the limited healthcare access of patients with autoimmune diseases during the COVID-19 pandemic. Consequently, alternative sample matrices are being explored for the measurement of autoantibodies. Our study aimed to establish the feasibility of measuring autoantibodies in saliva samples using a novel and highly sensitive method for the detection of autoantibodies. (2) Methods: A total of 48 serum/saliva pairs were collected and tested using a novel particle-based multi-analyte technology (PMAT) system for the presence of a wide range of autoantibodies. (3) Results: A high level of correlation was observed between the results obtained with serum and saliva (Spearman’s rho = 0.725). Study participants clearly preferred saliva over serum sampling as part of the usability assessment. (4) Conclusions: Saliva represents a promising alternative sample matrix for the detection of autoantibodies. The usability study showed a clear preference of saliva over serum as a sample matrix.

## 1. Introduction

Autoimmune diseases are characterized by autoantibodies directed to a large number of antigenic targets [1]. In systemic lupus erythematosus (SLE), for example, more than 180 different autoantibodies have been described [2]. Similarly, in many other autoimmune diseases, such as systemic sclerosis (SSc) or idiopathic inflammatory myopathies (IIM), a wide range of autoantibodies have been described. Although not all are clinically relevant and actionable, several are used for the diagnosis and management of patients with autoimmune diseases. Some of the most relevant antibodies include those to dsDNA, RNP, Sm, Ro52, Ro60, SS-B, CENP, Scl-70, Ribo-P, and Jo-1. Antibodies to dsDNA, Sm, and Ribosomal P are specific for systemic lupus erythematosus (SLE), anti-Scl70 and anti-CENP for systemic sclerosis (SSc), and anti-Jo-1 for idiopathic inflammatory myopathies (IIM). While antibodies to the other antigens (RNP, Ro52, Ro60, SS-B) are not specific for an individual disease, they are strongly associated with the presence of a systemic autoimmune disease [1]. In addition, more recently, antibodies to dense fine speckled antigen 70 (DFS70) have been described as a potential marker that helps to rule out antinuclear antibody (ANA)-associated rheumatic disease (AARD) when present in isolation (without accompanying antibodies to dsDNA, RNP, Sm, Ro52, Ro60, SS-B, CENP, Scl-70, Ribo-P, Jo-1). Historically, such tests were mostly ordered by rheumatologists to confirm a diagnosis of an underlying autoimmune disease. However, with the evolving concept of precision medicine (PM) inducing early diagnosis and intervention, novel approaches are required. In rheumatoid arthritis (RA) for example, several studies aimed to identify patterns of biomarkers that can help in the prediction of RA [3] with the intent to prevent overt disease development. In the context of autoimmune disease diagnostics, serum is the most commonly used sample matrix for the detection of autoantibodies. However, this sample type is dependent on venous puncture and requires medical personnel for sampling. Consequently, alternative sample types including capillary blood in the form of dried blood spots [4] or saliva have been proposed [5]. Saliva is a complex fluid secreted by the salivary glands and gingiva. It contains proteins (e.g., immunoglobulins, cytokines) as well as organic and inorganic substances that are important for maintaining oral health. Saliva may also be used for diagnostic purposes, offering clear advantages over other samples, including low-cost and non-invasive collection that can be performed by individuals with limited training. This is particularly valuable in children for whom blood collection may be challenging [5]. A limited number of studies have explored the feasibility of detecting autoantibodies in saliva [6,7,8,9,10,11,12,13,14,15]. The majority of studies focused on the detection of antibodies to citrullinated cyclic peptides (CCPs) which are a hallmark in the diagnosis of RA. This is of particular importance in light of the limited healthcare access of patients with autoimmune diseases during the COVID-19 pandemic. Our study aimed to establish the feasibility of measuring autoantibodies in saliva samples using a novel and highly sensitive method for the detection of autoantibodies. In addition, we studied patient preference of sample collection, comparing saliva to serum as the sample matrix.

## 2. Materials and Methods

Serum and saliva samples (paired specimens) were obtained from 48 consecutive unselected patients attending the outpatient’s clinic of tertiary referral center for autoimmune diseases for a follow-up appointment. All patients were diagnosed in concordance to the respective classification criteria. The main characteristics are shown in Table 1.

Saliva samples were collected using a saliva collection device as previously described [16]. In brief, saliva samples were collected by instructing participants to gently brush their gum line with an Oracol S14 saliva collection device (Malvern Medical Developments, Northbrook, Worcester, UK) for 1 to 2 min or until saturation. This saliva collection method specifically harvests saliva and gingival crevicular fluid (GCF), which is enriched with primarily IgG derived from serum [17]. The saturated sponge was then inserted into the storage tube, capped, and stored at 4 °C until processing whenever possible. Saliva was separated from the Oracol S14 swabs by centrifugation (10 min at 1500× *g*) and transferred into the attached 2 mL cryovial.

All samples were tested using a novel fully automated particle-based multi-analyte technology (PMAT, Inova Diagnostics, Werfen Autoimmunity, San Diego, CA, USA, research use only) which utilizes paramagnetic particles with unique signatures and a digital interpretation system, as previously described. In brief, antigens are coupled to paramagnetic particles that carry unique signatures and incubated with diluted patient samples. After 9.5 min of incubation at 37 °C, particles are washed and incubated for 9.5 min at 37 °C with anti-human IgG conjugated to phycoeryhtrin (PE) to label the bound autoantibodies. After the final wash cycle, fluorescent signal intensity on the particles is captured using a digital imager and analyzed using proprietary algorithms to extract meaningful information for each analyte. Saliva samples were tested at a final dilution of 1:10. The panel of autoantibodies comprised antibodies to dsDNA, RNP, Sm, Ro52, Ro60, SS-B, CENP, Scl-70, Ribo-P, Jo-1, and DFS70.

Results derived from PMAT for each analyte were assessed using median fluorescence intensity (MFI) values. To analyze the agreement between saliva and serum-based testing, receiver operating characteristics (ROC) curves were generated for each analyte which had 10 or more positive samples (RNP, Ro52, Ro60, and dsDNA). In addition, an analysis was conducted with all analytes pooled. Spearman’s coefficient was used to analyze the correlation between results obtained with serum vs. saliva. *p* values < 0.05 were considered significant. Data visualization was carried out using DataLab (Werfen, Barcelona, Spain).

Usability and patient preference were studied using a questionnaire of 10 questions in four categories: satisfaction, side effects, feasibility, and convenience (see Table 2) (modified from Vermersch et al. [17]). Patients were asked to provide answers for each item (ranging from 0 to 10), referring to the saliva test and the blood test, separately. The questionnaire was administered in patients’ local language (Italian). Differences were analyzed using a paired non-parametric test and differences <0.05 were considered significant.

## 3. Results

**Figure 1:**
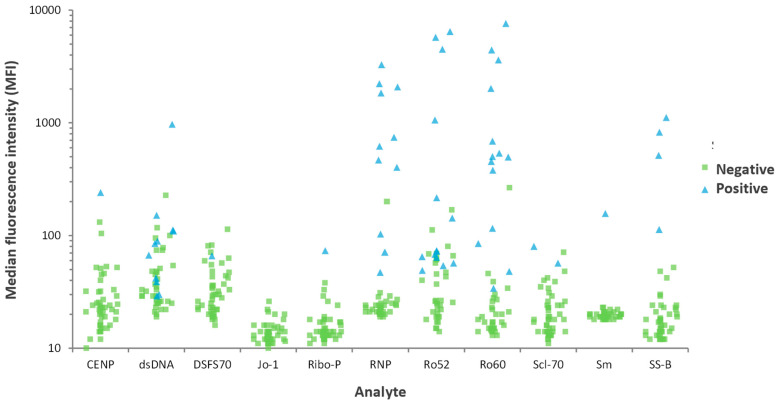
Scatter plot of autoantibodies measured in saliva samples stratified by antibody specificity. Patients are color coded based on the autoantibody status (antibodies to dsDNA, RNP, Sm, Ro52, Ro60, SS-B, CENP, Scl-70, Ribo-P, Jo-1, and DFS70) as defined using the serum sample (green = negative; blue = positive).

The testing of autoantibodies in saliva and the patient preference data are summarized in the following section. The highest number of positive samples (using serum) was found for Ro52 and Ro60 (n = 14) followed by RNP and dsDNA (n = 11), SS-B (n = 4), Scl-70 (n = 2), Sm, Ribo-P, DFS70, and CENP (n = 1). No positive case was found for Jo-1 (see Table 3). The levels of all autoantibodies tested are summarized in Figure 1.

The highest titers of antibodies were found for anti-RNP, anti-Ro52, anti-Ro60, and anti-SS-B.

### 3.1. Correlation between Results Obtained with Serum vs. Saliva

**Figure 2:**
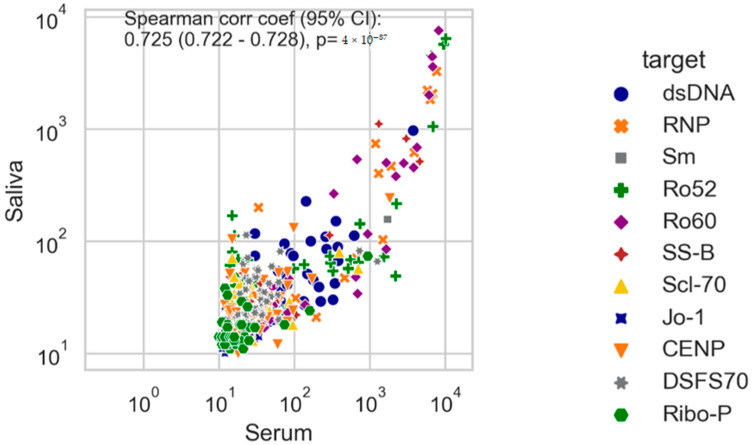
Autoantibodies levels measured in saliva in patients negative or positive based on the serum test results. The color code indicates the identity of the respective autoantibody (dsDNA, RNP, Sm, Ro52, Ro60, SS-B, CENP, Scl-70, Ribo-P, Jo-1). Correlation between serum and saliva. rho = 0.73 (95% CI 0.68–0.76), *p* < 0.0001. NOTE: *p* = 4 × 10^−87^ = *p* < 0.0001.

**Figure 3:**
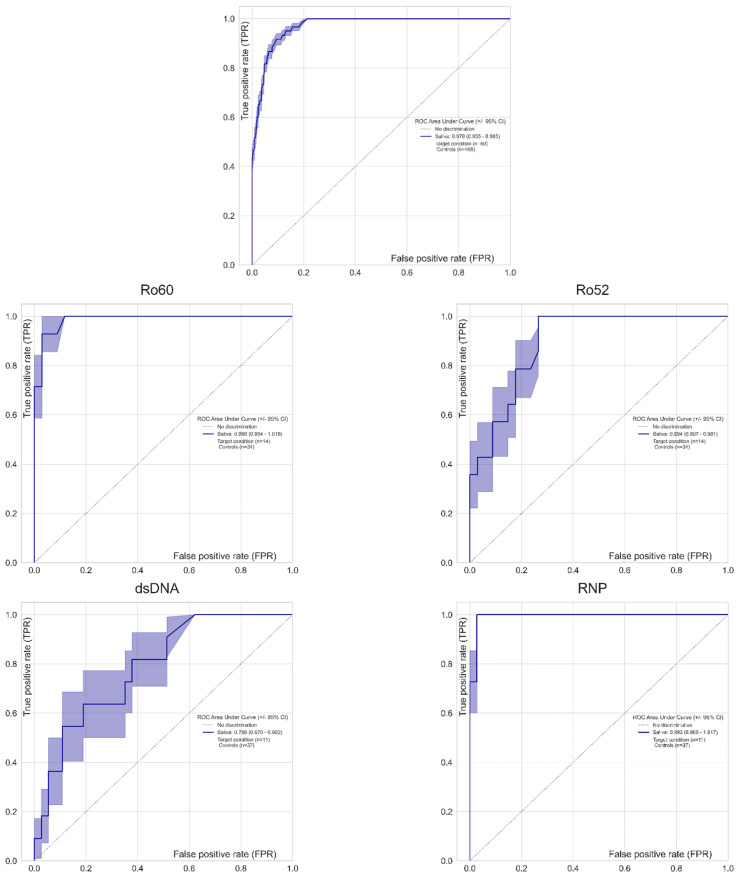
Receiver operating characteristic (ROC) analysis. The results obtained with saliva were used to generate a ROC curve using the serum results as a binary classifier. Results for all autoantibodies were analyzed pooled (all antibodies) or individually for RNP, Ro52, Ro60, and dsDNA. The results show a very high level of discrimination between serum positive and negative samples depending on the antibody titer defined using saliva samples. The area under the ROC curve was 0.97.

We observed a very high level of quantitative correlation between saliva and serum rho = 0.73 (95% CI 0.68–0.76, *p* < 0.0001) (see Figure 2). When the results were stratified in serum positive vs. serum negative samples, significantly higher results were observed in the saliva samples that corresponded to the positive serum samples. To assess the qualitative agreement between the two sample matrices irrespective of the threshold, we performed ROC analyses with the serum results as the binary classifier (see Figure 3). The combined analysis showed excellent discrimination between serum positive and negative samples with an area under the curve (AUC) of 0.97. In the subsets, significant differences were observed. While Ro60 and RNP showed excellent discrimination as well (both AUC of 0.99), a significantly lower discrimination could be observed for Ro52 (AUC = 0.89) and Jo-1 (AUC = 0.79).

### 3.2. Usability and Patient Preference of Sample Collection

**Figure 4:**
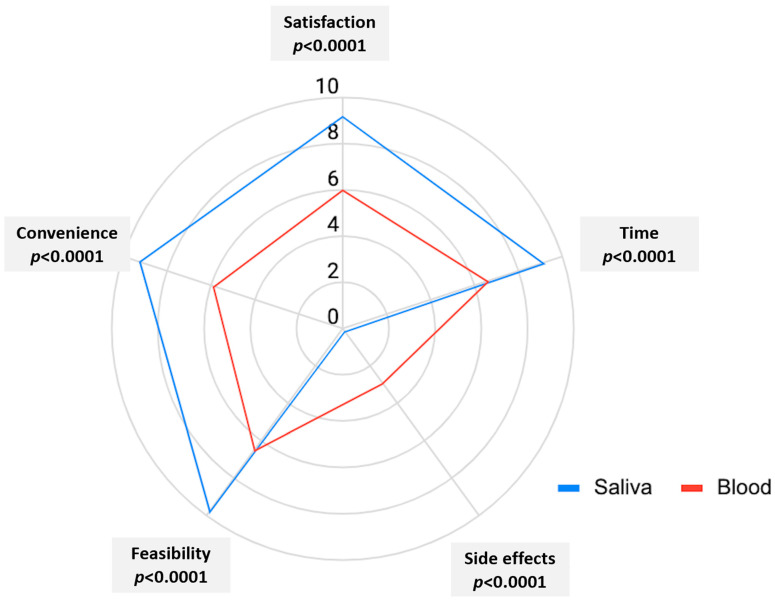
The radar chart showing the mean distributions of the scoring points for each item (satisfaction, time, feasibility, side effects, convenience). Results for all five parameters significantly favored saliva (*p* < 0.0001 for all).

As previously shown, we assumed the legitimacy of summing questionnaire item scores, without weighting or standardization, to generate domain scores [18]. Summing is considered legitimate when items of a domain are broadly parallel and contribute similarly to the construct being measured. These requirements are considered satisfied when items have similar means and variances. Data completeness was excellent (100%). With the above assumptions, we observed that the use of the saliva collection device was well accepted and preferred over serum collection by 96% of the participants. All factors favored saliva over serum as the sample matrix (*p* < 0.0001). The radar chart (see Figure 4) shows the mean distributions of the scoring points for each item (satisfaction, time, feasibility, side effects, convenience). Remarkably, all included patients reported the absence of any side effects (mean 0) for the use of the saliva device. Interestingly, for all domains, scores for the saliva test (but not blood) did not span the whole scale range, demonstrating skewing towards high scores.

No differences in terms of preferences were observed when dividing patients for age, sex, or diagnosis (data not shown).

## 4. Discussion

The detection of autoantibodies represents an important component in the diagnosis of several autoimmune diseases [1]. Despite significant efforts in the standardization of autoantibodies, major differences can still be observed, mostly depending on the analytes [19]. The COVID-19 pandemic has limited access to healthcare, especially for patients with chronic conditions such as autoimmune diseases. Consequently, alternative sample types that do not require visits to healthcare facilities have been studied and proposed. Such approaches include dried blood spots (DBS) [4] as well as recently described saliva [6,12] applications. A recent systematic literature review indicated a high correlation for most antibody testing areas using DBS. Consequently, a wide range of different collection devices have been developed and introduced into clinical practice [20,21]. Here we report the feasibility of the simultaneous measurement of autoantibodies in saliva samples, which could open new avenues for the diagnosis and follow-up of patients with autoimmune conditions. Human saliva is a complex fluid secreted by the salivary glands and gingiva. It contains proteins, including cytokines, as well as organic and inorganic substances that are important for maintaining oral health. Saliva may also be used for diagnostic purposes, offering clear advantages over other samples, including low-cost and non-invasive collection that can be performed by individuals with limited training. This is particularly valuable in children for whom blood collection may be challenging [5].

Several assays have been developed for the detection of antibodies to SARS-CoV-2 to assess vaccine response and/or past virus exposure. Based on the good performance of those assays, measurement of SARS-CoV-2-specific saliva antibodies should be considered as a complementary non-invasive assay to serum/plasma to determine COVID-19 prevalence and transmission in pediatric populations before and after vaccination campaigns [22,23,24,25,26,27,28].

In addition, a few pilot studies have been published that describe the measurement of autoantibodies in RA, pemphigus, SLE [6], primary biliary cirrhosis (PBC) [15], sicca syndrome, and concussion (as summarized in Table 4) [8]. The antibodies include anti-cyclic citrullinated peptide [9,10,11], anti-PAD4 [7], anti-desmoglein 1 and 3 [14], and anti-mitochondrial antibodies [15].

In one of the studies, anti-mitochondrial antibody type 2 (AMA-M2), a pivotal biomarker for the diagnosis of PBC, was simultaneously studied in saliva and serum. Both the presence and the titers of AMA-M2 were significantly associated with PBC. The correlation between saliva and serum was rho = 0.68. These findings indicated that saliva might be a less invasive and cost-effective medium to accurately test for AMA-M2 levels; this is a promising development for the diagnosis and monitoring of PBC. Most of the studies in RA focused on the correlation between autoantibodies and disease activity.

Of particular interest, a recent study evaluated salivary ANA using indirect immunofluorescence (IIF) and ELISA (IgG, IgA, and IgM). The levels of ANA were significantly higher in SLE patients compared with healthy controls. Salivary ANA was detected in 67.1% of SLE patients compared with 10.0% of healthy controls (*p* < 0.001). In addition to IgG, IgA and IgM antibodies were also able to discriminate SLE patients from controls, as shown by ROC analysis with AUC values of 0.72, 0.73, and 0.77, respectively. Interestingly, the ANA-IgM levels also correlated with disease activity as defined by the physician global assessment (PGA) and SLE disease activity index (SLEDAI), and negatively with serum C3 and C4. Salivary IgG-ANA also correlated with the erythrocyte sedimentation rate (ESR) and SLEDAI, and negatively with serum C3. Salivary ANA levels correlated with serum ANA titer, and salivary IgM-ANA and IgG-ANA correlated variably with PGA, SLEDAI, ESR, and complement levels. For the sensitivity detection of autoantibodies in saliva, low detection limits of the assay used are paramount. Although not part of this study, the detection limits of the PMAT assay have been defined and are summarized in Supplementary Table S1.

Our study expands on the data reported by Zhang et al. [6] and is the first to determine antinuclear antibodies (ANA) to a wide range of autoantigens (dsDNA, RNP, Sm, Ro52, Ro60, SS-B, CENP, Scl-70, Ribo-P, Jo-1, and DFS70) in serum and saliva pairs showing a high level of agreement between the sample matrices (rho = 0.73, 95% CI 0.68–0.76, *p* < 0.0001). Interestingly, the concordance between serum and saliva varied between the analytes. The ROC curves for RNP and Ro60 showed significantly higher concordance between the two matrix types compared with Ro52 and dsDNA, which is potentially linked to the antibody levels (amount of samples close to the decision point) and not to different IgG profiles in serum vs. saliva [29].

In the second part of our study, we analyzed the user preference, comparing saliva with the well-established serum matrix in terms of overall satisfaction, time, feasibility, side effects, and convenience. Remarkably, most of the patients clearly preferred saliva over serum for the majority of the criteria. However, it is important to consider that there are significant limitations of saliva as a sample matrix, including the variability of IgG content as well as the saliva secretion of patients. The latter becomes extremely important in the context of patients with SjS who exhibit significantly reduced saliva production.

Our study had several limitations that we want to point out. Due to the small number of patients, our study focuses on analytical analysis (main method of comparison for saliva vs. serum). In addition, we did not explore pre-analytical aspects of saliva as a matrix for antibody detection. Although several of those pre-analytical aspects have been studied [30], future studies are mandatory to also investigate clinical parameters next to pre-analytical aspects which include matrix stability, interference with food intake, etc. Lastly, cut-off values have to be established for the autoantibodies to nuclear antigens, as has been done for antibodies to pathogens [25].

## 5. Conclusions

Here we confirmed the ability to measure autoantibodies of the IgG isotype to nuclear autoantigens in saliva, which might represent a novel pathway for the detection of autoantibodies as part of the diagnosis of autoimmune conditions. These findings underscore the potential of using salivary autoantibody detection, particularly for future applications since saliva is easier to obtain than blood. The use of saliva as a sample matrix might open up new avenues for certain applications where serum samples are challenging to obtain. Such areas might include disease prediction and prevention in the era of precision medicine and precision health [31]. However, it is critical to consider the pre-test probability of a disease when deploying testing to larger groups of patients. This is especially important for tests with moderate specificity [1]. Therefore, for early diagnosis, modified cut-offs and proper triage systems are required. In addition, remote monitoring of disease activity, as shown in multiple sclerosis, could represent an effective approach to manage patients with autoimmune conditions [32].

## Figures and Tables

**Table 1:** Main characteristics and diagnoses of patients.

Demographics.	
Age, years [m ± sd]	51 ± 17.0
Females [n (%)]	38 (79.1%)
**Diagnosis**	
SLE [n (%)]	21 (43.8%)
PAPS [n (%)]	10 (20.8%)
SSc [n (%)]	2 (4.2%)
SjS [n (%)]	2 (4.2%)
UCTD [n (%)]	8 (16.7%)
Thyroiditis [n (%)]	1 (2.1%)
RA [n (%)]	4 (8.3%)

SLE–Systemic Lupus Erythematosus; PAPS–Primary Antiphospholipid syndrome; SSc–Systemic sclerosis; SjS–Sjögren’s syndrome; UCTD–Undifferentiated connective tissue disease; RA–Rheumatoid arthritis.

**Table 2:** Prevalence of autoantibodies in the patient cohort.

Satisfaction	1.	How satisfied or dissatisfied are you overall with the test? [0–10]
	2.	How satisfied or dissatisfied are you with the amount of time it takes the test to be administered? [0–10]
Side Effects	3.	As a result of being administered the test, do you currently/did you experience any side effects at all? YES/NO
	4.	How bothersome are the side effects of the testing you have been administered to monitor your condition? [0–10]
	5.	To what degree have the experienced side effects affected your overall satisfaction with the testing? [0–10]
Feasibility	6.	How easy or difficult is it to use the testing in its current form? [0–10]
	7.	How easy or difficult is it to plan when you will use the testing each time? [0–10]
Convenience	8.	How convenient or inconvenient is it to take the test as instructed? [0–10] Overall, how confident are you that being administered the testing in this form is a good thing for you? [0–10]
	9.	How certain are you that the good things about this testing outweigh the bad things? [0–10]

**Table 3:** Prevalence of autoantibodies in the patient cohort.

Antibody	No. (%) Serum Positive
dsDNA	11/48 (22.9%)
RNP	11/48 (22.9%)
Sm	1/48 (2.1%)
Ro52	14/48 (29.2%)
Ro60	14/48 (29.2%)
SS-B	4/48 (8.3%)
CENP	1/48 (2.1%)
Scl-70	2/48 (4.2%)
Ribo-P	1/48 (2.1%)
Jo-1	0/48 (0.0%)
DFS70	1/48 (2.1%)

**Table 4:** Overview of studies on autoantibodies measured in saliva.

Study	Disease	Reference
Demoruelle et al. 2021	RA	[7]
Roos Ljungberg et al. 2020	RA	[9]
Svärd et al. 2020	RA	[10]
Svärd et al. 2020	RA	[11]
Koopai et al. 2018	Pemphigus	[14]
Zhang et al. 2022	SLE	[6]
Lu et al. 2017	PBC	[15]
Burbelo et al. 2019	SS	[13]
Pin et al. 2021	C	[8]

## Data Availability

Data will be made available upon appropriate request.

## Supplementary Materials

The following supporting information can be downloaded at: https://www.mdpi.com/article/10.3390/diagnostics12082026/s1.
