# Supplementary material for: Detection of Autoantibodies in Saliva as New Avenue for the Diagnosis and Management of Autoimmune Patients
Source: Diagnostics (Basel). 2022 Aug 22;12(8):2026. doi: 10.3390/diagnostics12082026 (PMC9407454; doi:10.3390/diagnostics12082026)
Supplement: Supplementary file 1 [file diagnostics-12-02026-s001.zip › diagnostics-1760201-supplementary.pdf]

**Table S1.** Supplement figure Limit of blank (LoB), limit of detection (LoD), limit of quantitation (LoQ); MFI = median fluorescence intensities.

| Analyte | Final LoB MFI | Final LoD MFI | Final LoQ MFI |
|---|---|---|---|
| dsDNA | 19 | 33 | 41 |
| RNP | 21 | 24 | 24 |
| Sm | 20 | 25 | 26 |
| Ro52 | 15 | 19 | 26 |
| Ro60 | 15 | 19 | 24 |
| SS-B | 11 | 18 | 30 |
| Scl-70 | 11 | 16 | 22 |
| Jo-1 | 11 | 13 | 15 |
| Centromere | 11 | 18 | 35 |
| DFS70 | 21 | 31 | 45 |
| Ribo-P | 11 | 21 | 21 |
